# Sequential autologous CAR-T and allogeneic CAR-T therapy successfully treats central nervous system involvement relapsed/refractory ALL: a case report and literature review

**DOI:** 10.3389/fonc.2024.1341682

**Published:** 2024-01-23

**Authors:** Yifan Liu, Yanfen Li, Zhangyu Yu, Rongrong Wang, Yu Jing

**Affiliations:** Medical School of Chinese PLA, Department of Hematology in the Fifth Medical Center of PLA General Hospital, Beijing, China

**Keywords:** acute lymphoblastic leukemia, central nervous system, refractory and relapsed, car-t, neurotoxicity

## Abstract

**Background:**

The central nervous system (CNS) is the most common site of extramedullary invasion in acute lymphoblastic leukemia (ALL), and involvement of the CNS is often associated with relapse, refractory disease, and poor prognosis. Chimeric antigen receptor-T (CAR-T) cell therapy, a promising modality in cancer immunotherapy, has demonstrated significant advantages in the treatment of hematological malignancies. However, due to associated adverse reactions such as nervous system toxicity, the safety and efficacy of CAR-T cell therapy in treating CNSL remains controversial, with limited reports available.

**Case report:**

Here, we present the case of a patient with confirmed B-ALL who experienced relapse in both bone marrow (BM) and cerebrospinal fluid (CSF) despite multiple cycles of chemotherapy and intrathecal injections. The infusion of autologous CD19 CAR-T cells resulted in complete remission (CR) in both BM and CSF for 40 days. However, the patient later experienced a relapse in the bone marrow. Subsequently, allogeneic CD19 CAR-T cells derived from her brother were infused, leading to another achievement of CR in BM. Significantly, only grade 1 cytokine release syndrome (CRS) and immune effector cell-associated neurotoxicity syndrome (ICANS) events were detected during the treatment period and showed improvement with symptomatic management. During subsequent follow-up, the patient achieved a disease-free survival of 5 months and was successfully bridged to hematopoietic stem cell transplantation.

**Conclusion:**

Our study provides support for the argument that CNS involvement should not be deemed an absolute contraindication to CAR-T cell therapy. With the implementation of suitable management and treatment strategies, CAR-T therapy can proficiently target tumor cells within the CNS. This treatment option may be particularly beneficial for relapsed or refractory patients, as well as those with central nervous system involvement who have shown limited response to conventional therapies. Additionally, CAR-T cell therapy may serve as a valuable bridge to allogeneic hematopoietic stem cell transplantation (allo-HSCT) in these patients.

## 1 Introduction

**Figure 1:**
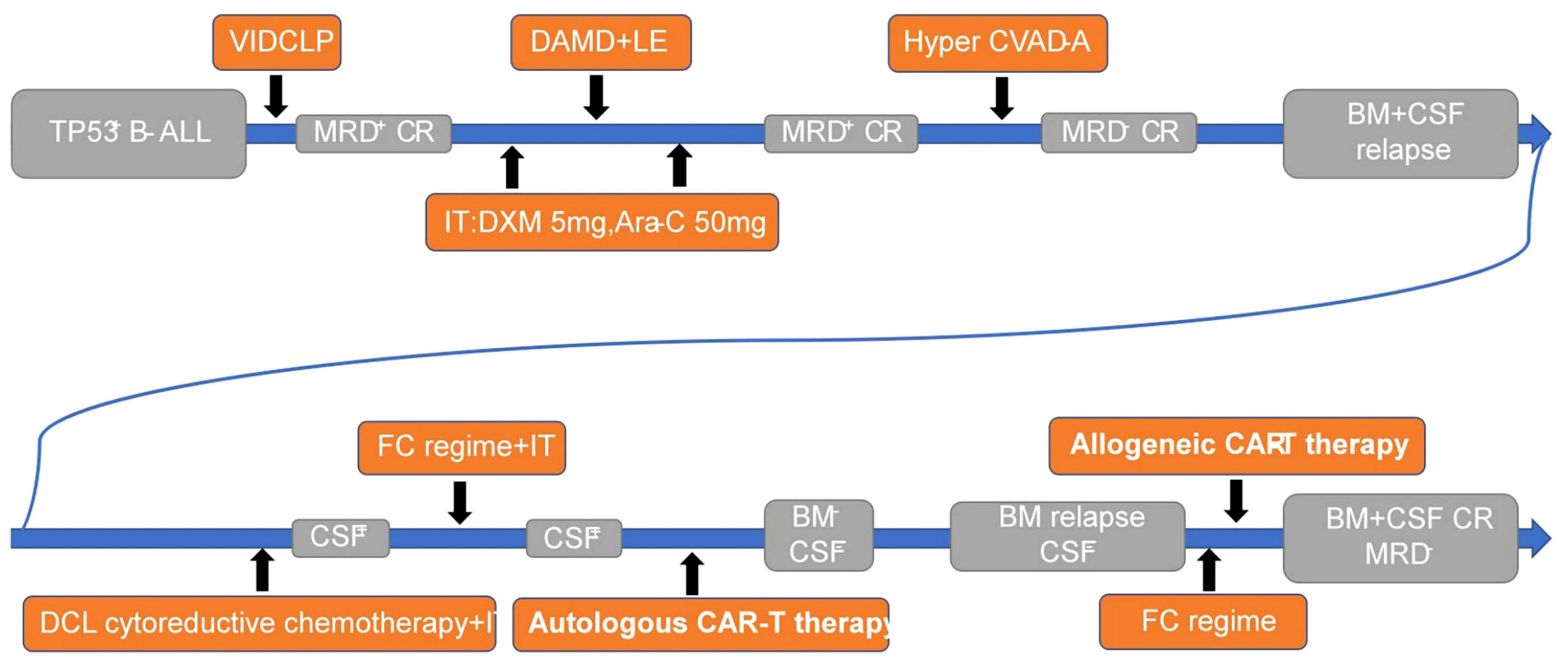
Summary protocol and response of the patient’s treatment.

Despite the central nervous system being the most common site of extramedullary invasion in acute lymphoblastic leukemia, current treatment options for central nervous system leukemia (CNSL) remain limited, resulting in poor outcomes that do not meet patient needs. In recent years, chimeric antigen receptor-T cell therapy has emerged as a prominent immunotherapy, offering high remission rates alongside significant side effects (1, 2). However, patients with CNSL are often excluded from CAR-T therapy due to concerns that the toxic effects may exacerbate symptoms (3). Consequently, the use of CAR-T therapy for CNSL remains a topic of controversy, with a lack of relevant studies available. This article presents a singular case study of TP53 gene-mutated B-cell ALL, wherein the patient experienced bone marrow and cerebrospinal fluid relapse despite multiple chemotherapy regimens and intrathecal injections. Autologous CD19 CAR-T cell infusion was administered, leading to complete remission of both CSF and BM for a period of 40 days. However, subsequent BM relapse occurred. Subsequently, allogeneic CAR-T cells were infused, resulting in another achievement of complete remission in BM. Notably, mild adverse reactions were observed throughout the entire CAR-T cell therapy process (**Figure 1**). Additionally, a follow-up monitoring of five months demonstrated disease-free survival, providing evidence for the feasibility of CAR-T cell therapy for CNSL.

## 2 Leukemia chemotherapy process

### 2.1 Background

A 23-year-old woman began experiencing symptoms of dizziness and fatigue, which gradually worsened over time. She sought medical attention at a local hospital due to persistent fever, with temperatures fluctuating between 38.0°C and 39.0°C. Upon initial diagnosis, blood routine examination revealed the following results: hemoglobin level of 83 g/L, platelet count of 89×10^9/L, and white blood cell count of 15.7×10^9/L. Bone marrow examination showed blast cells accounting for 45.2% of the total cell population, leading to a diagnosis of B-cell acute lymphoblastic leukemia. Further immunophenotyping of leukemia cells revealed blast cells comprising 58.90% of the population, predominantly expressing CD10, CD19, CD20, CD24, cCD22, CD38, and HLA-DR, while partially expressing CD34. Genetic testing indicated that 44.74% of the blast cells carried TP53 gene mutations and 20.47% carried PIGA gene mutations, while no fusion genes were detected. This patient was diagnosed with high-risk B-ALL with TP53 mutation, a genetic subtype typically associated with poor prognosis. Subsequently, the patient was transferred to the First Medical Center of PLA General Hospital for further management and treatment. Lumbar puncture was performed to assess the cerebrospinal fluid, which showed no noticeable abnormalities.

### 2.2 The first course of chemotherapy

We implemented a decitabine + VICLP regimen as the initial course of induced remission chemotherapy. The specific dosage and administration were as follows: Dexamethasone 5mg d1; Vindesine 4mg d1/d8/d15; Idarubicin 10mg d1/d8/d15; Cyclophosphamide 1g d1, 0.6g d15; PEG-ASP 3750iu d9; Methylprednisolone 40mg d8-d22; Decitabine 10mg d14-d20. After completing this course of treatment, a bone marrow reexamination was conducted to evaluate the therapeutic effect. The results demonstrated that the average proportion of immature lymphocytes in the bone marrow was 4.0%, and residual leukemia cells of 1.82% were detected through leukemia immunophenotyping. These findings indicated that the chemotherapy regimen employed in this course of treatment was effective, leading to complete remission in the patient. However, measurable residual diseases (MRD) testing yielded positive results.

### 2.3 The second course of chemotherapy

Following the successful response to the initial course of chemotherapy, we administered decitabine + adolescent acute lymphoblastic intensive therapy as the second course of consolidation and intensive treatment. The specific dosage and administration were as follows: Decitabine 10mg d1-d5; Cytarabine 2.5g d1-d2 Q12h; Dexamethasone 10mg d1-d2 Q12h; PEG-ASP 3750iu d16; Methotrexate 4.5g d15, 30mg d27; Vindesine 4mg d16; 6-Mercaptopurine 75mg d16-d22; Cyclophosphamide 600mg d26-d27, Etoposide 100mg d26-d27. Additionally, lumbar puncture was performed to evaluate the CSF, and intrathecal injection of chemotherapy drugs (Dexamethasone 5mg and Cytarabine 50mg) was administered to prevent the occurrence of CNSL both before and after this course of treatment. The CSF examination revealed no abnormalities, and the bone marrow examination indicated that immature lymphocytes accounted for 2.0%. However, residual leukemia cells of 1.18% were still detected through immunophenotyping. Although the patient achieved complete remission in the bone marrow, MRD remained positive following this course of treatment.

### 2.4 The third course of chemotherapy

Despite the previous two courses of chemotherapy, the patient continued to exhibit positive MRD in the bone marrow, indicating refractory bone marrow involvement. To address this, we opted for the Hyper CVAD-A consolidation chemotherapy regimen as the third stage of treatment. The specific dosage and administration were as follows: Cyclophosphamide 0.4g d1-d2 Q12h; Vindesine 4mg d1,d11; Doxorubicin 40mg d4; Dexamethasone 20mg d1-d4, 20mg d11-d14. Following the completion of this course of treatment, the results indicated that blast cells accounted for approximately 2% of the bone marrow, and no residual tumor cells were detected through leukemia immunophenotyping. Furthermore, the proportion of TP53 gene mutation decreased to 4.123%. After this course of treatment, the patient achieved a negative MRD status in the bone marrow.

### 2.5 Occurrence of CNSL

Following three different chemotherapy regimens, the patient achieved MRD negative CR in the bone marrow. Due to the high expression of CD19 on tumor cells as revealed by immunophenotyping, our plan was to proceed with CD19-targeting CAR-T therapy as a bridging strategy and followed by hematopoietic stem cell transplantation. However, approximately two weeks later, a bone marrow examination revealed that primitive immature lymphocytes accounted for 40% of the bone marrow, indicating relapsed bone marrow manifestations of acute lymphoblastic leukemia. As a result, we promptly implemented cytoreductive chemotherapy to reduce the tumor burden while preparing for CAR-T therapy. Concurrently, lumbar puncture was performed to evaluate the cerebrospinal fluid, and intrathecal injection of chemotherapy drugs (Dexamethasone 5mg and Cytarabine 50mg) was administered. Unfortunately, the CSF analysis indicated the presence of naive B cells with abnormal phenotypes (2.45×10^2 cells/ml), suggesting infiltration of leukemia cells into the central nervous system.

To summarize, despite receiving three rounds of chemotherapy and two courses of intrathecal preventative chemotherapy, the patient experienced relapse in both the CNS and BM shortly after achieving CR. This signifies the development of relapsed and refractory CNSL. Consequently, determining the next appropriate treatment plan poses a significant challenge.

## 3 CAR-T cell therapy process

### 3.1 The first CAR-T cell treatment

After thorough evaluation and analysis of the patient’s condition, including the central nervous system and other systems, it was determined that she was still suitable for CAR-T immunotherapy. Following a comprehensive understanding of the benefits, drawbacks, potential complications, and adverse reactions associated with CAR-T therapy, the patient provided voluntary informed consent and was enrolled in a clinical study on CD19 CAR-T cell therapy (IRB approval number: S2019-217-01). Anti-CD19 CAR-T cells were generated using a lentiviral vector derived from fresh leukapheresis materials obtained from the patient. T cells were activated and transduced with a lentiviral vector containing the anti-CD19 CAR gene, which consists of the CD19 recognition domain, hinge domain, transmembrane domain, CD137/4-1BB intracellular domain, and CD3ζ intracellular domain. The transduced CAR-T cells were then cultured in medium supplemented with 300 IU/mL IL-2 at 37°C/5% CO2 for approximately 5–11 days to obtain sufficient cells for infusion. Real-time quantitative polymerase chain reaction (PCR) was utilized to quantify the level of the CAR gene, with less than 100 copies/μg DNA considered negative. Flow cytometry (FCM) was performed to determine the transduction efficiency and the ratio of B cells in the peripheral blood following CAR-T cell infusion.

Prior to CAR-T cell infusion, intrathecal injection (Dexamethasone 5mg, Cytarabine 50mg, and Methotrexate 10mg) and lymphodepletion using the fludarabine-cyclophosphamide regimen (Fludarabine 30mg/m^2×4d and Cyclophosphamide 500mg/m^2×2d) were administered. Due to the impact of high-intensity chemotherapy on the quality of peripheral blood mononuclear cells (PBMCs) in this patient, T cell expansion during CAR-T cell manufacturing was unsatisfactory. Therefore, a relatively low dose of 0.73×10^5/kg CAR-T cells(the CAR-T cells were expanded *in vitro* from their own peripheral blood T cells for 8d), manufactured by Beijing Yongtai Biological Products Co., Ltd., was infused into the patient. Vital signs and inflammatory indicators were closely monitored following the infusion of CAR-T cells.

**Figure 2:**
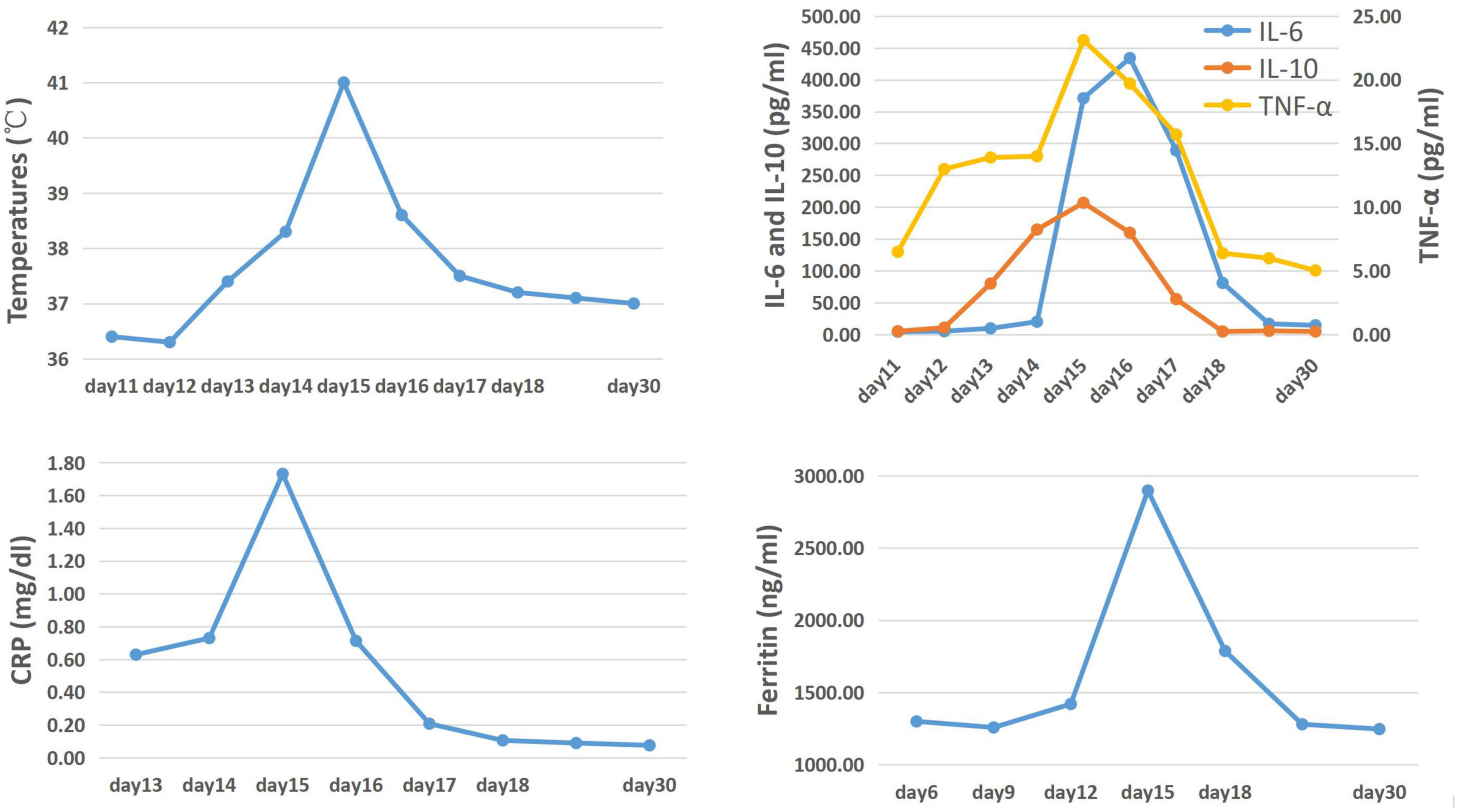
The trend of temperature, cytokines, CRP and ferritin levels after the first CAR-T cells infusion.

During the first 13 days following the infusion of CAR-T cells, no side effects were observed in the patient. However, on the 14th day after infusion, the patient developed a fever and showed an upward trend in temperature. Despite this, there were no significant fluctuations in inflammatory indicators (**Figure 2**). The following day, the temperature continued to rise, and there was a significant increase in inflammatory factors such as IL-6 and CRP, indicating the occurrence of cytokine release syndrome associated with CAR-T cell infusion. In addition to physical cooling and antibiotics for treating infection, Tocilizumab 240mg (5mg/kg) was administered to control CRS. On the 16th day after infusion, although the patient’s temperature began to decrease, she experienced persistent severe headaches, and the detected IL-6 index continued to increase, suggesting the occurrence of CRS in the CNS. To alleviate symptoms while minimizing the negative impact on the proliferation and function of CAR-T cells, Dexamethasone 5mg was administered, resulting in rapid relief of symptoms within a few hours. In the subsequent days, both inflammatory markers and temperature started to decline and returned to normal levels by the 18th day after infusion (**Figure 2**), indicating successful passage of the side effect stage after CAR-T treatment.

**Figure 3:**
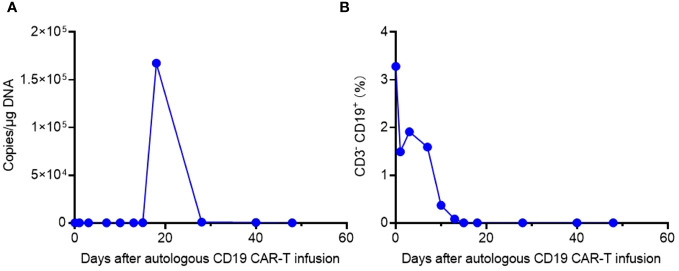
The *in vivo* kinetics of CAR-T cells and dynamics of CD3- CD19+ target cells. **(A)** Expansion and persistence of CAR-T cells shown by the CAR gene copies per μg DNA in the patient’s peripheral blood. **(B)** Dynamics of CD3- CD19+ target cells in the patient’s peripheral blood detected by flow cytometry.

Laboratory data also revealed the *in vivo* kinetics of CAR-T cells and the dynamics of CD3- CD19+ target cells (**Figure 3**). Expansion of CAR-T cells commenced on Day 15 and reached its peak on Day 18 after infusion (**Figure 3A**), which correlated with the clinical symptoms observed in the patient. CSF and BM examinations conducted on Day 20, Day 25, and Day 30 after CAR-T cell infusion did not detect any residual tumor cells. Consistent with these findings, CD3- CD19+ target cells were not detected in the peripheral blood after Day 15 (**Figure 3B**). These results indicate the effectiveness of CAR-T cell therapy, with the patient achieving MRD-negative complete remission. However, there was a significant decrease in CAR gene copies on Day 28, suggesting relatively poor persistence of CAR-T cells (**Figure 3A**). Subsequent BM examination on Day 40 revealed relapse.

### 3.2 The second CAR-T cell treatment

On Day 40 after the initial CAR-T cell infusion, bone marrow examination revealed an increase in primitive lymphocytes to 16%, and flow cytometry detected 4.05% residual leukemic cells, indicating a relapse of leukemic cells in the bone marrow. However, no abnormalities were found in the CSF through flow cytometry analysis. The relapse may be attributed to the poor persistence of autologous CAR-T cells, potentially resulting from the impaired proliferative capability of T cells following long-term and high-intensity chemotherapy. Consequently, consideration was given to allogeneic CAR-T cell therapy using cells derived from the patient’s brother.

**Figure 4:**
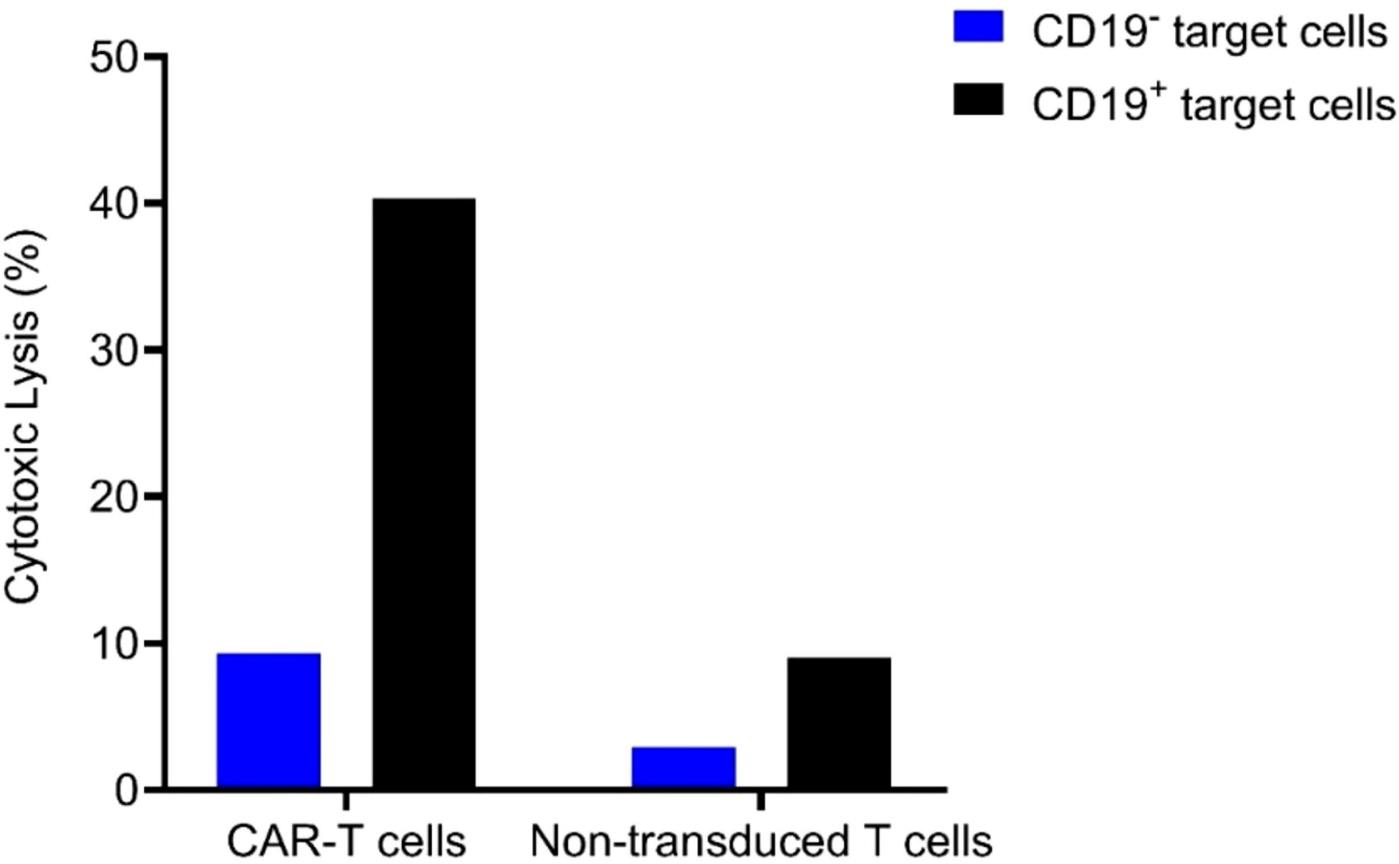
Cytolytic activity of the allogeneic CAR-T cells and its non-transduced T cells control when the E (effector) to T (target) ratio is 3:1.

Comparing the patient and her brother’s HLA loci, it was determined that they were fully matched, allowing for the safe infusion of allogeneic CAR-T cells. As an adjuvant drug, the Bruton’s tyrosine kinase (BTK) inhibitor ibrutinib was employed in conjunction with CAR-T cells to enhance therapeutic efficacy. Prior to allogeneic CAR-T cell infusion, the FC regimen was administered for lymphocyte depletion as per routine practice. The patient’s brother was healthy and his T cells expanded well *in vitro* during CAR-T manufacture. Considering that the infused allogeneic T lymphocytes may cause the risk of graft-versus-host disease (GVHD), we chose a moderate dose of 1.48×10^6/kg CAR-T cell infusion (the CAR-T cells were expanded *in vitro* from the patient’s brother peripheral blood T cells for 6d). Laboratory data confirmed the high anti-tumor efficacy of the CAR-T cells *in vitro* (**Figure 4**).

**Figure 5:**
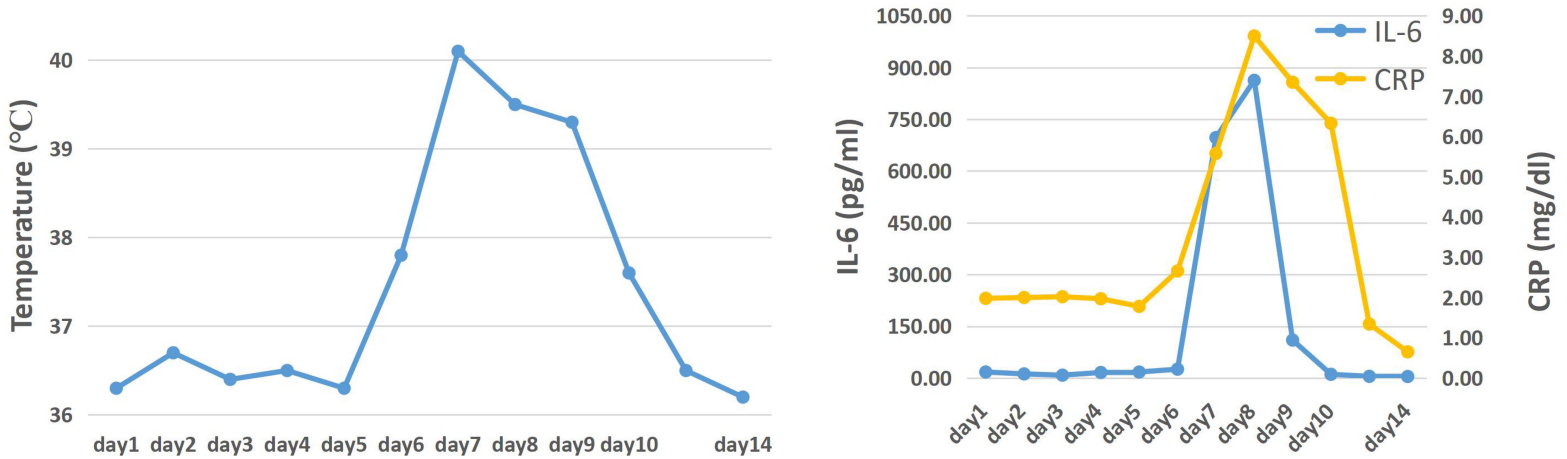
The trend of temperature, IL-6 and CRP levels after the second CAR-T cells infusion.

During the first five days post-infusion, the patient did not experience any side effects. On Day 6, she developed a fever, which was monitored closely and managed with physical cooling and antibiotic treatment. The following day, the temperature further increased, accompanied by significant elevations in IL-6 and CRP levels (**Figure 5**), indicative of CRS reaction. Treatment with Tocilizumab 240mg was initiated. Steroids was not administered this time to avoid potential interference with CAR-T cell expansion. Over the subsequent days, careful monitoring of the patient’s temperature and inflammatory markers revealed a gradual decrease starting on Day 8 post-infusion (**Figure 5**), with no development of discomfort.

**Figure 6:**
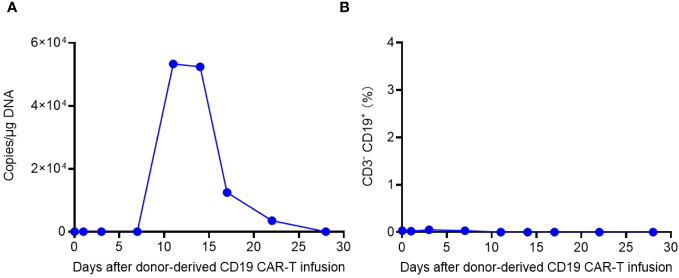
The *in vivo* kinetics of donor-derived CAR-T cells and dynamics of CD3- CD19+ target cells. **(A)** Expansion and persistence of CAR-T cells shown by the CAR gene copies per μg DNA in the patient’s peripheral blood. **(B)** Dynamics of CD3- CD19+ target cells in the patient’s peripheral blood detected by flow cytometry.

The *in vivo* kinetics of CAR-T cells and dynamics of CD3- CD19+ target cells were depicted in **Figure 6**. CAR-T cell expansion reached its peak on Day 11 and exhibited a slower decline compared to the allogenic infusion. Bone marrow examinations conducted on Days 14 and 20 after allogeneic CAR-T cell infusion confirmed CR and MRD negativity, indicating the effectiveness of allogeneic CAR-T treatment. Consistent with these results, CD3- CD19+ target cells were not detected in the peripheral blood after Day 11 (**Figure 6B**).

### 3.3 Summary

In summary, despite undergoing multiple chemotherapy regimens and autologous CAR-T cell therapy, the patient experienced bone marrow relapse, indicating a persistent and refractory tumor manifestation. In light of the poor response to previous treatments, allogeneic CAR-T cell therapy was chosen, leading to complete remission within a relatively short period. The two CARs that patients receive have second-generation structures, using a 4-1BB-derived costimulatory domain and CD3ζ-derived activation domain. The CD3+CAR+ percentage among the autologous and donor-derived CAR-T infusion products are 49.5% and 41.96%, respectively. The viability of the two infusion products were 85.9% and 88.89%, respectively. In addition to cell number, viability and CD3+ CAR+, negative results for sterility, mycoplasma and endotoxin and other factors were also included in releasing criteria.

At the time of writing, the patient has a disease-free survival of five months and has been successfully bridged to hematopoietic stem cell transplantation in MRD-negative remission. Notably, the patient did not experience severe toxic side effects throughout the treatment process, with only Grade 1 ICANS and mild myelosuppression observed. The myelosuppression was successfully managed through red blood cell transfusion and granulocyte-colony stimulating factor (G-CSF). No significant neurological toxicity was observed. Therefore, the overall treatment proved to be effective and demonstrated a favorable safety profile.

## 4 Discussion and literature review

Acute lymphoblastic leukemia is a common malignant tumor in the blood system, with an incidence of nearly 30% in adult leukemia, but it ranks first in childhood tumors (4). Although the mature application of systematic chemotherapy regimens and hematopoietic stem cell transplantation technology in recent years has greatly improved the remission rate and survival rate of ALL (5), there are still facing great challenges for R/R ALL, especially CNSL patients, and there is no unified treatment plan (6–8). Such patients not only have a poor prognosis but also prone to metastases in the central nervous system or other systems (9). Especially when the central nervous system is involved, which often represents a worse prognosis (10). According to literature reports, the five-year survival rate of CNSL does not exceed 30%, and the median survival time is only 6 months (11, 12). At present, there are limited treatments and efficacy for CNSL. Most medical centers prevent the development of central metastases through the early use of intrathecal chemotherapy. When CNSL occurs, it is mainly through increasing the frequency of intrathecal injections or using high-dose systemic chemotherapy with chemotherapeutic drugs that can cross the blood-brain barrier. Furthermore, whole-brain and whole-spinal cord radiation therapy may present as an alternative, however, it may result in heightened neurological symptoms such as cognitive deterioration and memory impairment. And even after receiving systemic high-dose chemotherapy and cranial radiotherapy, nearly 8% of CNSL still relapse (13), so there is an urgent need for more effective treatments for CNSL (14, 15).

CAR-T cell therapy uses gene editing technology to transform immune T cells into CAR-T cells that can specifically recognize tumor cells, so it can play a precise anti-tumor effect and has played a huge advantage in the treatment of hematological malignancies (16, 17). For example, CD19 CAR T-cell have demonstrated CR rates of 70~90% in patients with relapsed/refractory B-cell acute lymphoblastic leukemia (r/r B-ALL) (18–20). Studies have confirmed that CAR-T cells can cross the blood-brain barrier (BBB) and enter CNS to play a role (21), providing a theoretical basis for CAR-T therapy for the treatment of CNSL (22). However, due to the particularity of the central nervous system and CAR-T cell therapy-related neurotoxicity, CNS invasion is often regarded as a contraindication for CAR-T therapy in clinical studies (3, 23). Therefore, it is still very controversial whether CAR-T therapy can be the treatment choice for CNSL.

Clinical reports have demonstrated that anti-CD19 CAR-T cells can effectively eliminate leukemia cells from CSF with fully reversible toxicity, indicating that patients with r/r B-ALL and CNS involvement may benefit from CAR-T cell therapy with an acceptable safety profile. A successful case of CAR-T therapy for CNSL was published by the Hematology Department of Southwest Hospital in August 2021 (24). The patient, initially diagnosed with B-ALL, developed CNS involvement after VDLP chemotherapy. Subsequent treatment with a Cytarabine + VDLP regimen and intrathecal injections (Cytarabine, Methotrexate, and Dexamethasone) failed to eradicate residual leukemia cells in the CNS. Consequently, the patient received anti-CD19 CAR-T cell infusion to treat CNSL. On the fourth day after CAR-T cell infusion, the patient experienced grade 1 CRS symptoms, such as headache, vomiting, high fever, and increased inflammatory markers. These symptoms were successfully managed with tocilizumab and mannitol. By the seventh day, the patient’s vital signs and various indicators returned to normal. After one month, both the bone marrow and CSF achieved CR. It is worth pointing out that the patient still obtained a disease-free follow-up period of up to 36 months without further chemotherapy and stem cell transplantation, indicating a remarkable efficacy of the treatment.

In another retrospective study published by the Children’s Hospital of Philadelphia in August 2021 (1), 195 patients with ALL or lymphocytic lymphoma who underwent CAR-T therapy were analyzed. These patients were divided into two groups based on CNS involvement to compare treatment responses. The results indicated no significant differences in the incidence of CR, relapse-free survival (RFS), overall survival (OS), and CRS between the CNS+ and CNS- groups. However, the incidence of NT was higher in the CNS+ group compared to the CNS- group (58% vs. 41%), primarily driven by a higher incidence of grade 1 NT. Notably, in observations with a median follow-up of 39 months, isolated CNS+ patients receiving CAR-T therapy exhibited significantly higher 2-years overall survival rates than CNS+ combined with bone marrow positivity patients and CNS- patients (91% vs. 71% vs. 71%). These findings suggest that CNS involvement alone should not be considered an absolute contraindication to CAR-T therapy. In fact, in cases of isolated CNS involvement, CAR-T therapy may even be preferred due to the relatively better treatment response.

Although the anti-tumor ability of CAR-T cells has been widely validated through a large number of studies, the inability of CAR-T cells to exhibit durability remains one of the major challenges to this therapy. The potential reasons for poor persistence primarily include T cell exhaustion: T cells may enter a state of exhaustion due to prolonged exposure to high-intensity stimulation (which may arise from sustained tumor antigen presentation or excessive cytokine release), leading to diminished differentiation capacity and anti-tumor effects; cell memory and differentiation state: T cells extracted from patients may exhibit differences in their differentiation state, which could impact their *in vivo* persistence (19, 25, 26). Relatively “young” central memory T cells (TCM) and stem cell memory T cells (TSCM) may possess better self-renewal and long-term survival capabilities, whereas more “mature” effector memory T cells (TEM) may have poorer persistence; immune evasion: Cancer cells may evade recognition by CAR-T cells through altering surface antigens or other mechanisms, including downregulation or lack of antigen expression, or expression of immune checkpoint molecules that inhibit T cell activation; other potential reasons may include the influence of the tumor microenvironment, CAR-T cell manufacturing process, or construct design. Numerous studies are currently striving to address this issue, such as utilizing memory T cell subtypes, combining immune modulators, or enhancing T cell homing effects (27, 28).

In our study, patients received sequential treatment with DVICLP, decitabine-adolescent acute intensive regimen, and Hyper CVAD-A regimen chemotherapy, along with multiple intrathecal injections of chemotherapeutic agents. Nevertheless, tumor recurrence in the bone marrow and central nervous system still occurred. However, after treatment with autologous CAR-T cells, complete remission was achieved in both the bone marrow and CSF within forty days. Although there was another bone marrow relapse, subsequent allogeneic CAR-T therapy led to rapid complete remission and consistently negative CSF results. Importantly, no serious complications were observed throughout the entire treatment process, indicating that the safety and efficacy of the treatment met expectations.

## 5 Conclusions

At present, it is premature to definitively conclude the safety and efficacy of CAR-T cell therapy in the treatment of CNSL. Further retrospective analysis and prospective studies with larger sample sizes are needed to confirm its effectiveness and safety. However, based on our limited experience and existing clinical studies (29–31), it appears that CNS invasion should not be considered an absolute contraindication to CAR-T therapy. In cases where standard therapies have failed for patients with relapsed and refractory disease or isolated CNS involvement, CAR-T cell therapy may be a viable treatment option. Despite the limited *in vivo* sustained expansion of CAR-T cells currently, their potent short-term anti-tumor effect presents an opportunity to establish favorable conditions for subsequent allogeneic hematopoietic stem cell transplantation. This approach could serve as an effective transitional option for patients scheduled to undergo allo-HSCT.

## Data availability statement

The original contributions presented in the study are included in the article/Supplementary Material. Further inquiries can be directed to the corresponding author.

## Ethics statement

The studies involving humans were approved by General Hospital of the Chinese People’s Liberation Army. The studies were conducted in accordance with the local legislation and institutional requirements. The participants provided their written informed consent to participate in this study. The manuscript presents research on animals that do not require ethical approval for their study. Written informed consent was obtained from the individual(s) for the publication of any potentially identifiable images or data included in this article.

## Author contributions

YiL: Methodology, Project administration, Writing – original draft, Writing – review & editing, Data curation. YaL: Data curation, Project administration, Software, Supervision, Writing – original draft. ZY: Formal analysis, Writing – original draft, Data curation, Project administration. RW: Data curation, Formal analysis, Project administration, Software, Writing – original draft. YJ: Formal analysis, Funding acquisition, Methodology, Resources, Supervision, Validation, Writing – original draft, Writing – review & editing.
